# The Expression Level of *BRCA2* and Its Changes during Chemotherapy in Patients with Different Pathological Types of Mammary Cancer

**Published:** 2019-09

**Authors:** Yu HUANG, Min LUO, Junqing HUANG, Shaoxin HUANG, Liuxia WEI, Yumei ZHANG, Zhiming ZHANG

**Affiliations:** Department of Medical Oncology, Affiliated Tumor Hospital of Guangxi Medical University, Nanning 530021, P.R. China

**Keywords:** Noninvasive mammary cancer, Invasive mammary cancer, *BRCA2*, Chemotherapy

## Abstract

**Background:**

We aimed to investigate the expression level of breast cancer susceptibility gene 2 (*BRCA2*) and its changes during chemotherapy in patients with different pathological types of mammary cancer (MC).

**Methods:**

Overall, 102 patients treated in Affiliated Tumor Hospital of Guangxi Medical University, China from April 2013 to August 2017 were enrolled as experimental group, including 58 patients with noninvasive MC (group A) and 44 with invasive MC (group B). Fifty healthy volunteers at the same time were enrolled as control group. The relative expression of *BRCA2* in the blood of MC patients was detected by real-time fluorescence quantitative PCR (FQ-PCR).

**Results:**

In the experimental group, the expression level of *BRCA2* in group A was higher than that in group B before chemotherapy (*P*<0.001); the expression level in group A and group B 1 month after chemotherapy was higher than that before chemotherapy (*P*<0.001); the expression level in the both groups 3 months after chemotherapy was higher than that 1 month after chemotherapy (*P*<0.001); the expression level of *BRCA2* in blood of group A increased gradually before, 1 month and 3 months after chemotherapy (*P*<0.001). The expression level of *BRCA2* in blood of group B increased gradually at the same time points (*P*<0.001).

**Conclusion:**

*BRCA2* is over-expressed in noninvasive MC patient and under-expressed in invasive MC patient. And it can be used as an index for monitoring the condition of MC patients with different pathological types during chemotherapy.

## Introduction

Mammary cancer (MC) is one of the most common malignant tumors in women in the world, ranking first in the incidence of female malignancies. According to the WHO in 2016, about 1.263 million women are diagnosed with MC every year, and 278000 people die from the disease (1). Since 2010, the incidence of MC has increased by more than 22%, the mortality rate has increased by more than 13%, and there is an obvious upward trend, especially in the fast-paced developed countries and large cities (2). MC has the characteristics of high incidence, high number of new cases, high mortality rate and low diagnostic rate, which seriously endangers the health of women and consumes a large amount of health resources every year (3).

In recent years, modern medical technology in China has been continuously developed and improved in the treatment of MC, but the disease monitoring and 5-year survival rate of patients with MC have not been substantially improved (4). Therefore, it is of great clinical significance and value to explore the occurrence and development mechanism of MC, and to find out an effective MC disease monitor.

Breast cancer susceptibility gene 2 (*BRCA2*) is mapped on 13q12, and has 27 exons and encodes a protein containing 3418 amino-acid residues, and has 8 internal repeats known as BRC sequences. *BRCA2* protein is located in the nucleus and often related to the repair of DNA (5). *BRCA2* expression is not only associated with the incidence of MC and ovarian cancer, but also affects the incidence of pancreatic cancer and prostate cancer (6). At present, it is believed that the variation of *BRCA2* may lead to the decline in the stability of *BRCA2* protein structure, promote the abnormal proliferation of cells, prevent the normal differentiation of the cells and eventually lead to the occurrence of the tumor, which is one of the mechanisms that lead to the occurrence of MC (7). This gene is a tumor suppressor gene that encodes tumor suppressor protein and inhibits tumor growth by regulating tumor production (8).

Therefore, the expression of *BRCA2* in serum of patients with different pathological types of MC and the changes of *BRCA2* expression in blood before and after chemotherapy were discussed in this study to explore the monitoring value of *BRCA2* in patients with different pathological types of MC during chemotherapy, and to provide reference for clinical practice.

## Materials and Methods

### Clinical data

Overall, 102 pathologically confirmed MC patients treated in Affiliated Tumor Hospital of Guangxi Medical University, China from April 2013 to August 2017 were randomly selected as experimental group, with an average age of (57.4±11.6) years. According to different pathological types, the experimental group was divided into two subgroups: noninvasive MC patients (group A, n=58) and invasive MC patients (group B, n=44). At the same time, 50 healthy volunteers with an average age of (55.1±14.2) years undergoing physical examination in our hospital were collected as control group. This study was approved by the Ethics Committee of the Institute, and all subjects were informed and agreed to participate in the clinical study and signed fully informed consent.

### Inclusion and exclusion criteria

Inclusion criteria: Patients diagnosed with MC by clinicopathological diagnosis in our hospital; Age≥18 yr; No congenital hereditary disease; No radiotherapy, chemotherapy and other anti-cancer treatment before sampling.

Exclusion criteria: Patients who had taken antibiotics within 3 months before sampling; patients with liver insufficiency; patients with autoimmune system deficiency; patients with other tumors; patients with cardiovascular disease; patients with recurrent MC.

### Main reagents and instruments

EDTA anticoagulant tube (Beijing Biocoen Biotechnology Co., Ltd, 366450). Human peripheral blood lymphocyte separating medium (Shanghai Beinuo Biotechnology Co., Ltd.). Reverse transcription system (Applied Biological Systems Inc, USA.). FQ-PCR reaction system (Applied Biological Systems Inc, USA.). GAPDH contrast kit (Shanghai Jianglai Biotechnology Co., Ltd, 48T/96T). MC chemotherapeutic drug Taxol (Bristol-Myers Squibb Company, USA, Registration number: X19990416). MC chemotherapeutic drug Carboplatin (Bristol-Myers Squibb Company, USA, Registration number: H20110231). The Taqman probe sequence was synthesized by primer express 5.0 software of PE Company. cDNA standard was synthesized by Shanghai Shenyou Co., Ltd. The primer sequences of *BRCA2* and GAPDH were designed and synthesized by Suzhou Syn-Biotechnology Co., Ltd. See Table 1.

**Table 1:** Gene sequence primer table of BRCA2

***Groups***	***Upstream primer***	***Downstream primer***
BRCA2	5′-CTTGCCCCTTTCGTCTATTTG-3′	5′-TACGGCCCT-GAAGTACAGTCTT-3′
GAPDH	5′—ACCACAGTCCATGCCATCAC—3′	5′—TCCACCACCCTGTTGCTGTA—3′

### Methods

Collection of specimens: The experimental steps were carried out under aseptic conditions. Five ml elbow vein blood of all subjects was taken on an empty stomach in the morning, placed in the EDTA anticoagulant vacuum tube, slowly and evenly stirred, and stored at −20 °C or in liquid nitrogen for use.

Chemotherapy of MC, Taxol (Bristol-Myers Squibb Pharmaceutical Co., Ltd. USA, Registration number: X19990416) and Carboplatin (Bristol-Myers Squibb Pharmaceutical Co., Ltd. USA, Registration number: H20110231) were used for MC chemotherapy. All patients in the experimental group took 20 mg Dexamethasone (Guangdong Huanan Pharmaceutical Group Co., Ltd, SFDA Approval No. H44024469) orally 6–12 hours before Taxol injection to prevent severe allergies. Carboplatin was given at 60 mg/kg of body weight, diluted with 0.9% normal saline injection, and light-shielded infused protected from light infusion. 14 days for 1 cycle, a total of 6 cycles of chemotherapy was performed. On the 1st/5th/10th day of the cycle, 60 mg/kg carboplatin was instilled. On the first day of the cycle, 175 mg/m^2^ Taisol was injected intravenously, and the injection was finished in 3 hours.

Determination of *BRCA2* expression: Blood samples were removed from liquid nitrogen and diluted with 0.9% saline (1:1), then 2 ml separating medium was added into the mixture After 20 min of centrifugation, the second layer of annular milky white lymphocytes from the top to bottom of the centrifuge tube was placed in the test tube containing normal saline, centrifuged for 20 min, precipitated and washed twice to obtain the required lymphocytes. The total RNA was extracted by one step method of guanidinium isothiocyanate - phenol - chloroform. Synthesis of cDNA was conducted after RNA extraction, with 20 μl reverse transcription system, including 1 μg total RNA, 200 ng random primer, 20U RNasin, 4 μl 5×reverse transcription reaction buffer, 20U M-MLV reverse transcriptase at 42 °C for 55 minutes. PCR: 5 μl reaction system reverse transcription product, 400 nmol/L oligonucleotide primer, 150 nmol/L probe, d ATP, d CTP and d GTP 200 μmol/L each, 400 μmol/L d UTP, 5 μl 10×buffer solution, l25 mmol/L Mg C, 1.25U TaqDNA polymerase, 0.5U Uracil N-glycosylase (UNG). The contents of *BRCA2* and GAPDH in standards and samples were determined by Sequence Detector analyzer. Amplification parameters: 40 cycles of 50 °C for 2 min, 95 °C for 10 min, 94 °C for 30 s, 66 °C for 1 min. After adjusting the threshold with Bio-Rad real-time quantitative PCR analysis software, the data were examined and analyzed to acquire the Ct value of each sample reaction, and the ΔCt wad calculated. ΔCt =Ct value of gene to be tested - Ct value of internal reference gene (GAPDH). Then compared with the control group, the data of 2-ΔCt were obtained and statistically analyzed.

### Statistical methods

SPSS17.0 (Shanghai Yuchuang Network Technology Co., Ltd.) was used for statistical analysis. The expression of *BRCA2* was expressed as mean ± standard deviation (x̄±s); T test was used to compare the measurement data between groups; chi-square test was used to compare the enumeration among groups. When *P*<0.05, the difference was statistically significant.

## Results

### Baseline data in experimental group and control group

There was no significant difference in age, fertility, amenorrhea, menarche time, smoking, body mass index (BMI) and other general clinical baseline data between the two groups. There were significant differences in tumor diameter, lymph node metastasis, skin viscosity and tumor differentiation degree and other pathological features between the subgroups (group A and group B) of the experimental group (*P*<0.05) (Table 2).

**Table 2:** Baseline data in experimental group and control group [n (%)]

***Clinical features***		***Experimental group (n=102)***		***Control group (n=50)***	***X^2^***	***p***
		**Group A (n=58)**	**Group B (n=44)**			
Age					0.342	0.843
	<55	18(31.03)	13(29.55)	13(26.00)		
	≥55	40(68.97)	31(70.45)	37(74.00)		
Amenorrhea					0.538	0.746
	Yes	51(87.93)	39(88.64)	42(84.00)		
	No	7(12.07)	5(11.36)	8(16.00)		
Smoking					0.596	0.742
	Yes	3(5.17)	2(4.55)	4(8.00)		
	No	55(94.83)	42(95.45)	46(92.00)		
BMI (kg/m^2^)					0.466	0.792
	<24	21(36.21)	16(36.36)	21(42.00)		
	≥24	37(63.79)	28(63.64)	29(58.00)		
Menarche time (Years)					0.633	0.729
	<15	44(75.86)	34(77.27)	41(82.00)		
	≥15	14(24.14)	10(22.73)	9(18.00)		
Fertility					0.518	0.772
	Yes	51(87.93)	39(88.64)	46(92.00)		
	No	7(12.07)	5(11.36)	4(8.00)		
Tumor diameter					7.037	0.013
	<3cm	48(82.76)	26(59.09)			
	≥3cm	10(17.24)	18(40.91)			
Lymph node metastasis					9.896	0.002
	No	44(75.86)	20(45.45)			
	Yes	14(24.14)	24(54.55)			
Skin viscosity					9.930	0.002
	No	34(58.62)	12(27.17)			
	Yes	24(41.38)	32(72.73)			
Tumor differentiation degree					7.834	0.009
	Medium and low	27(37.93)	29(65.91)			
	High	36(62.07)	15(34.09)			

### Detection of BRCA2 expression in serum of experimental group and control group

**Fig. 1:**
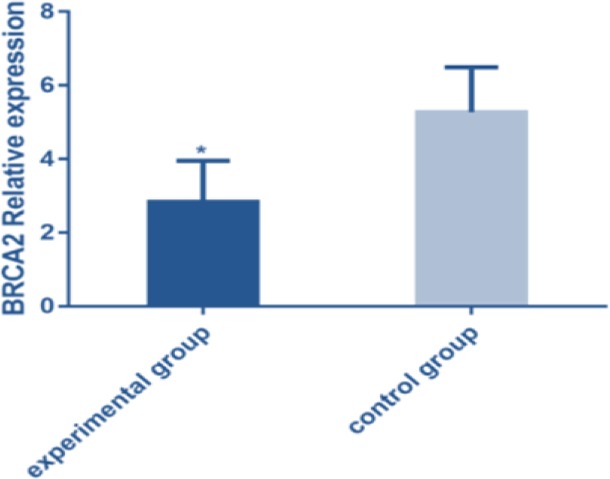
The expression of *BRCA2* in experimental group was lower than that in control group detected by FQ-PCR (*P*<0.001). Note: * represented that the difference between the experimental group and the control group was significant (*t*=20.17, *P*<0.001)

*BRCA2* was under-expressed in the blood of the experimental group (2.83±1.42) and was over-expressed in the blood of the control group (8.27±1.82). There was statistical difference between the two groups (*t*=20.17 *P*<0.001) (Fig. 1).

### Detection of BRCA2 expression in experimental group with different clinical features

The expression of *BRCA2* was not statistically different from age, fertility, amenorrhea, menarche time, smoking, BMI between the two groups. There were significant differences in the expression of *BRCA2* with tumor diameter, lymph node metastasis, skin viscosity and tumor differentiation degree (*P*<0.001) (Table 3).

**Table 3:** Expression of BRCA2 in experimental group with different clinical characteristics [n(%)]

***Clinical features***		***Number (n=102)***	***BRCA2***	***^2^/T***	***p***
Age (yr)				0.732	0.466
	<55	31	2.68±1.27		
	≥55	71	2.89±1.36		
Amenorrhea				0.527	0.599
	Yes	90	2.70±1.29		
	No	12	2.91±1.34		
Smoking				0.424	0.672
	Yes	5	2.65±1.24		
	No	97	2.91±1.34		
BMI (kg/m^2^)				0.347	0.729
	<24	37	2.88±1.37		
	≥24	65	2.61±1.20		
Menarche time (Years)				1.450	0.150
	<15	78	2.73±1.32		
	≥15	24	3.16±1.09		
Fertility				0.817	0.416
	Yes	90	2.79±1.38		
	No	12	3.13±1.12		
Tumor diameter				6.802	<0.001
	<3cm	74	3.11±1.14		
	≥3cm	28	1.63±0.22		
Lymph node metastasis				8.522	<0.001
	No	64	3.15±1.10		
	Yes	38	1.62±0.21		
Skin viscosity				3.838	<0.001
	No	46	3.09±1.16		
	Yes	56	2.26±0.85		
Tumor differentiation				7.388	<0.001
	Medium and low	51	1.93±0.52		
	High	51	3.17±1.08		

### Detection of expression of BRCA2 in experimental group with different pathological types before chemotherapy

**Fig. 2:**
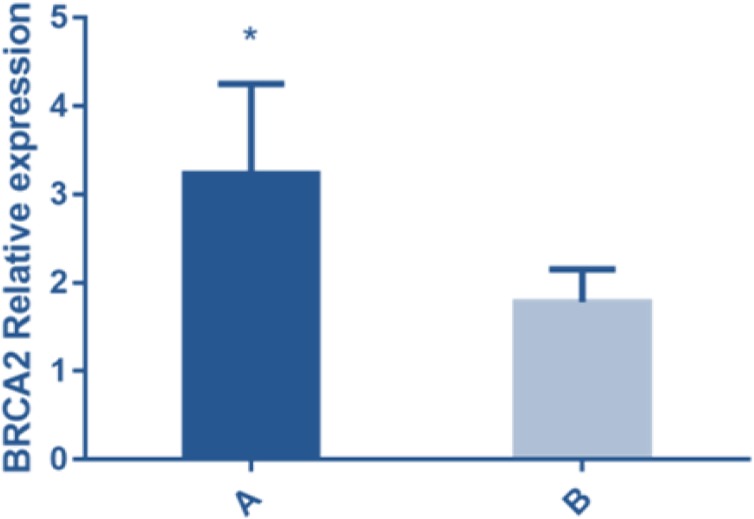
The expression of *BRCA2* in subgroups (Group A and group B) of the experimental group detected by FQ-PCR showed that the expression in the blood of group A was higher than that of group B (*P*<0.001). Note: * represented that the difference between the group A and group B was significant (*t*=8.983, *P*<0.001)

According to different pathological types, the experimental group was divided into two subgroups: noninvasive MC patients (group A, n=58) and invasive MC patients (group B, n=44). The *BRCA2* was under-expressed in group B before chemotherapy (1.78±0.37) and the expression of *BRCA2* in group A (3.23±1.02) was higher than that in group B, there was statistical difference between the two groups (*t*=8.983, *P*<0.001) (Fig. 2).

### Expression of BRCA2 in subgroups (Group A and group B) of the experimental group after chemotherapy

The expression of *BRCA2* in the group A before, 1 month and 3 months of chemotherapy showed an increasing trend, and the difference was statistically significant (*P*<0.001); the expression of *BRCA2* in group B at the same time points showed an increasing trend, and the difference was statistically significant (*P*<0.001) (Table 4).

**Table 4:** Expression of BRCA2 in subgroups (Group A and group B) of the experimental group after chemotherapy

***Chemotherapy time***	***Before chemotherapy***	***1 month after chemotherapy***	***3 months after chemotherapy***	***F***	***p***
Group A (n=58)	3.23±1.02	4.17±0.88^a^	5.31±0.79^ab^	77.400	<0.001
Group B (n=44)	1.78±0.37	2.89±0.72^a^	3.39±0.66^ab^	82.16	<0.001

Note: Compared with before chemotherapy,

a *p* < 0.001; compared with 1 month after chemotherapy,

b *p*<0.01

### Multivariate logistic regression analysis in the experimental group

Further multivariate analysis was performed on variables that were meaningful by univariate analysis, and the results showed that tumor diameter, lymph node metastasis, skin viscosity and tumor differentiation degree were selected as regression models (*P*<0.05). Tumor diameter, lymph node metastasis and skin viscosity were the risk factors affecting the expression level of *BRCA2*, and tumor differentiation was the protective factor (Table 5 and Table 6).

**Table 5:** Variable and assignment

***Variable***	***Assignment***
Tumor diameter (cm)	<3: 1
	≥3: 2
Lymph node metastasis	No: 1
	Yes: 2
Skin viscosity	No: 1
	Yes: 2
Tumor differentiation degree	Medium and low: 1
	High: 2
Constant	BRCA2 over-expression (2.21–4.25): 1
	BRCA2 under-expression (1.41–2.15): 2

**Table 6:** Multivariate unconditional logistic regression analysis

***Variable***	***β***	***p***	***X^2^***	***OR***	***95%CI***
Constant	−2.972	0.005	8.035	0.051	
Tumor diameter	0.850	0.042	4.145	2.339	1.032–5.298
Lymph node metastasis	1.091	0.004	8.296	2.977	1.417–6.253
Skin viscosity	1.365	0.002	9.879	3.916	1.672–9.172
Tumor differentiation	−1.384	<0.001	13.123	0.250	0.118–0.530

## Discussion

With the development of economy and the deterioration of environment, the incidence of various kinds of malignant tumors is increasing. MC, one of the most common malignant tumors in women especially in urban women, is the major diseases threatening the health of women and has a high incidence and morbidity rate (9). Its incidence is second only to lung cancer (10), and is rising year by year, becoming the fastest (11) in all malignant tumors. The MC patients have a poor prognosis and a low 5-year survival rate (12), and there is no good disease monitoring indicator for evaluation of the status of chemotherapy. The occurrence, development and metastasis of MC has a great correlation with oncogene and tumor suppressor genes (13). Therefore, exploring the mechanism of the occurrence and development of MC and finding effective indicators of MC disease monitoring indicator is the focus of clinical research at the present stage.

At present, epidermal growth factor receptor (EGFR) (14) is a common MC disease monitoring indicator, which has a unstable expression.

Some studies have shown that the expression of EGFR in MC may also be related to the decrease of degradation after activation, and the deletion of some domains of EGFR can lead to the destruction of receptor downregulation mechanism, activation of abnormal signal transduction pathway, inhibition of apoptosis and so on (15). Tumor marker 153 (CA153) is also known as one of the disease monitoring indicators (16), however, it lacks sufficient specificity and sensitivity, and has no obvious changes in serum of early MC patients, which is often similar to other benign diseases (17). Therefore, finding a stable, effective and sensitive MC disease monitor is a hot spot in the field of medicine.

*BRCA2*, the second MC susceptibility gene (18) found in recent years, is expressed in many tissues, and the expression is the highest in mammary gland and thymus and slightly lower in lung, ovary and spleen (19). Normal *BRCA2* protein is located in the nucleus and involved in the repair of DNA. The expression pattern of *BRCA2* gene is similar to that of breast cancer susceptibility gene 1 during cell amplification, that is, the transcription of *BRCA2* gene cannot be detected in resting cells (20). The expression of *BRCA2* in the rapidly proliferating cells increase significantly and has cell cycle dependent (21). These results suggest that *BRCA2* plays an important role in the regulation of cell growth.

By comparing the expression of *BRCA2* between the experimental group and the control group, it was found that *BRCA2* was under-expressed in the serum of the experimental group and over-expressed in the serum of the control group. The experimental group was divided into two groups (group A and group B) according to pathological classification. The comparison between the two groups found that the expression of *BRCA2* in the serum of noninvasive MC in group A was higher than that of invasive MC in group B. The expression of *BRCA2* in serum of group A and group B 1 month after chemotherapy was higher than that before chemotherapy. The expression of *BRCA2* in serum of the both groups 3 months after chemotherapy was higher than that 1 month after chemotherapy. Further multivariate analysis showed that tumor diameter, lymph node metastasis and skin viscosity were the risk factors affecting the expression level of *BRCA2*, and tumor differentiation was the protective factor. The results showed that the high expression level of *BRCA2* was associated with high tumor differentiation and small tumor diameter, and the low expression level of *BRCA2* with low tumor differentiation and large tumor diameter.

The results of Kong et al (22) suggested that the increased mutation rate of BRCA2 increased the susceptibility to breast, ovarian and prostate cancer, which indicates that that BRCA2 is involved in the development of cancer cells in patients with MC, and has some value in the monitoring of MC. The subjects in this study were screened strictly according to the inclusion and exclusion criteria, and the chemotherapeutic drugs of MC were the products of Bristol Myers Squibb, which ensured the reliability of the results. However, there are still some defects in this study. For example, there were fewer patients included in the study; more MC patients with different pathological types should be collected; the relationship between BRCA2 and other clinical symptoms in MC patients was not further studied in this study. However, we will improve the study by following up the patients regularly according to the data of the patients in the experimental group and analyzing the results after the test.

## Conclusion

Compared with normal volunteers, *BRCA2* is under-expressed in the blood of noninvasive MC patients and invasive MC patient. The expression level of *BRCA2* increases with the increase of chemotherapy time in MC patients. *BRCA2* can be used as a monitoring indicator for patients with different pathological types of MC during chemotherapy, which is worthy of widespread promotion in clinical practice.

## Ethical considerations

Ethical issues (Including plagiarism, informed consent, misconduct, data fabrication and/or falsification, double publication and/or submission, redundancy, etc.) have been completely observed by the authors.

## References

[1] Zou Z Hu J Mccoy TP (2014). Quality of life among women with breast cancer living in Wuhan, China. Int J Nurs Sci, 1: 79–88.

[2] Kim Y Yoo KY Goodman MT (2015). Differences in incidence, mortality and survival of breast cancer by regions and countries in Asia and contributing factors. Asian Pac J Cancer Prev, 16: 2857–70. 25854374 10.7314/apjcp.2015.16.7.2857

[3] Campbell I Scott N Seneviratne S Kollias J Walters D Taylor C Roder D (2015). Breast cancer characteristics and survival differences between Maori, Pacific and other New Zealand women included in the Quality Audit program of Breast Surgeons of Australia and New Zealand. Asian Pac J Cancer Prev, 16: 2465–2472. 25824782 10.7314/apjcp.2015.16.6.2465

[4] Schedin P (2006). Pregnancy-associated breast cancer and metastasis. Nat Rev Cancer, 6:281–91. 16557280 10.1038/nrc1839

[5] Lara K Consigliere N Pérez J Porco A (2012). BRCA1 and BRCA2 mutations in breast cancer patients from Venezuela. Biol Res, 45:117–30. 23096355 10.4067/S0716-97602012000200003

[6] Kasi PM Pedersen KS Mcwilliams RR (2015). *BRCA2*-associated pancreatic cancer and current screening guidelines. Cancer, 121: 3046. 26018189 10.1002/cncr.29447

[7] Doss CGP Nagasundaram N (2014). An Integrated in Silico Approach to Analyze the Involvement of Single Amino Acid Polymorphisms in FANCD1/*BRCA2*-PALB2 and FANCD1/*BRCA2*-RAD51 Complex. Cell Biochem Biophys, 70: 939–956. 24817641 10.1007/s12013-014-0002-9

[8] Tacconi EM Lai X Folio C (2017). BRCA1 and *BRCA2* tumor suppressors protect against endogenous acetaldehyde toxicity. EMBO Mol Med, 9: 1398–1414. 28729482 10.15252/emmm.201607446 PMC5623864

[9] Wen D He Y Wei L (2016). Incidence rate of female breast cancer in urban Shijiazhuang in 2012 and modifiable risk factors. Thorac Cancer, 7: 522–529. 27766774 10.1111/1759-7714.12357 PMC5130316

[10] Gradishar WJ Anderson BO Balassanian R (2015). NCCN Clinical Practice Guidelines in Oncology: Breast Cancer. Version 2.2015. J Natl Compr Canc Netw, 13: 448–475. 25870381 10.6004/jnccn.2015.0060

[11] Othieno-Abinya NA Nyabola LO Abwao HO Ndege P (2002). Postsurgical management of patients with breast cancer at Kenyatta National Hospital. East Afr Med J, 79(3): 156–62. 12389963 10.4314/eamj.v79i3.8897

[12] Xu J Yang W Wang Q (2014). Decreased HCRP1 expression is associated with poor prognosis in breast cancer patients. Int J Clin Exp Pathol, 7: 7915–7922. 25550832 PMC4270550

[13] Muggerud AA Rønneberg JA Wärnberg F (2010). Frequent aberrant DNA methylation of ABCB1, FOXC1, PPP2R2B and PTEN in ductal carcinoma in situ and early invasive breast cancer. Breast Cancer Res, 12(1): R3. 20056007 10.1186/bcr2466 PMC2880421

[14] Wolff AC Hammond MEH Hicks DG (2013). Recommendations for Human Epidermal Growth Factor Receptor 2 Testing in Breast Cancer: American Society of Clinical Oncology/College of American Pathologists Clinical Practice Guideline Update. J Clin Oncol, 31: 3997–4013. 24101045 10.1200/JCO.2013.50.9984

[15] Giró-Perafita A Palomeras S Lum D (2016). Preclinical Evaluation of Fatty Acid Synthase and EGFR Inhibition in Triple Negative Breast Cancer. Clin Cancer Res, 22: 4687–4697. 27106068 10.1158/1078-0432.CCR-15-3133

[16] Zheng H Luo RC (2005). [Diagnostic value of combined detection of TPS, CA153 and CEA in breast cancer]. Di Yi Jun Yi Da Xue Xue Bao, 25(10):1293–4, 1298 [In Chinese]. 16234113

[17] Yip CH Bhoo Pathy N Teo SH (2014). A review of breast cancer research in malaysia. Med J Malaysia, 69:8–22. 25417947

[18] Norquist B Harrell M Walsh T (2014). Germline mutations in cancer susceptibility genes in brca1 and *BRCA2* negative families with ovarian and breast cancer. Gynecol Oncol, 135: 383.

[19] Mcallister KA Haugen-Strano A Hagevik S (1997). Characterization of the rat and mouse homologues of the *BRCA2* breast cancer susceptibility gene. Cancer Res, 57: 3121–3125. 9242436

[20] Lou DI Mcbee RM Le UQ (2014). Rapid evolution of BRCA1, and *BRCA2* in humans and other primates. BMC Evol Biol, 14: 155. 25011685 10.1186/1471-2148-14-155 PMC4106182

[21] Chodosh LA (1998). Expression of BRCA1 and *BRCA2* in normal and neoplastic cells. J Mammary Gland Biol Neoplasia, 3: 389–402. 10819533 10.1023/a:1018784031651

[22] Shahid T Soroka J Kong E (2014). Structure and mechanism of action of the *BRCA2* breast cancer tumor suppressor. Nat Struct Mol Biol, 21: 962–968. 25282148 10.1038/nsmb.2899 PMC4222816

